# Saliva levels of Abeta1-42 as potential biomarker of Alzheimer's disease: a pilot study

**DOI:** 10.1186/1471-2377-10-108

**Published:** 2010-11-03

**Authors:** Felix Bermejo-Pareja, Desiree Antequera, Teo Vargas, Jose A Molina, Eva Carro

**Affiliations:** 1 Neurology Service, Hospital 12 de Octubre, Madrid, Spain; 2 Neurodegenerative Diseases Biomedical Research Center (CIBERNED), Madrid, Spain; 3 Neuroscience Laboratory, Research Center, Hospital 12 de Octubre, Madrid, Spain

## Abstract

**Background:**

Simple, non-invasive tests for early detection of degenerative dementia by use of biomarkers are urgently required. However, up to the present, no validated extracerebral diagnostic markers for the early diagnosis of Alzheimer disease (AD) are available. The clinical diagnosis of probable AD is made with around 90% accuracy using modern clinical, neuropsychological and imaging methods. A biochemical marker that would support the clinical diagnosis and distinguish AD from other causes of dementia would therefore be of great value as a screening test. A total of 126 samples were obtained from subjects with AD, and age-sex-matched controls. Additionally, 51 Parkinson's disease (PD) patients were used as an example of another neurodegenerative disorder. We analyzed saliva and plasma levels of β amyloid (Aβ) using a highly sensitive ELISA kit.

**Results:**

We found a small but statistically significant increase in saliva Aβ_42 _levels in mild AD patients. In addition, there were not differences in saliva concentration of Aβ_42 _between patients with PD and healthy controls. Saliva Aβ_40 _expression was unchanged within all the studied sample. The association between saliva Aβ_42 _levels and AD was independent of established risk factors, including age or Apo E, but was dependent on sex and functional capacity.

**Conclusions:**

We suggest that saliva Aβ_42 _levels could be considered a potential peripheral marker of AD and help discrimination from other types of neurodegenerative disorders. We propose a new and promising biomarker for early AD.

## Background

With increasing life expectancy across the world, Alzheimer's disease (AD), the most common cause of dementia, is a rapidly growing socioeconomic and medical problem. AD diagnosis is time consuming and requires a combination of clinical assessment, psychological testing, imaging and exclusion of other neurological disorders. In light of these facts, a molecular biomarker that could identify and classify AD would be particularly useful in order to confirm the diagnosis, to perform epidemiological screening, to identify distinct groups of patients, to predict the outcome of the disease, and to monitor its progression and its sensitivity to treatment. In fact, lack of tools to detect preclinical AD has been suggested to be one of the main obstacles for the development of new treatments [1]. The ideal biomarker for AD should detect a fundamental feature of neuropathology: it should be as sensitive and specific as the clinical diagnosis, reliable, reproducible, simple to perform, inexpensive and non-invasive (studies on blood, urine, saliva, or buccal scrapings). Moderately invasive tests (skin, rectal biopsies, bone marrow samples, or cerebrospinal fluid -CSF-) or cerebral biopsy, are inconvenient for routine clinical practice.

In humans, in addition to the classical accumulation in the brain, amyloid-beta protein (Aβ) deposits are found in peripheral regions, including skin [2], nasal mucosa [3], and the lacrimal [4], and lingual glands [5]. The use of human salivary gland biopsies has been recently described as a tool for research on familial amyloidotic polyneuropathy (FAD) [6], and on AD [7] because both amyloid precursor protein (APP) and Aβ are expressed in human salivary epithelial cells [7]. Saliva is produced from salivary glands and mucous membranes and, as a biological fluid, is simple to obtain. Additionally, salivary levels may reflect changes in CSF [8]. Recent studies showed association of activity and levels of salivary acetylcholinesterase (AChE) with AD [9]. These findings may prove to be a useful marker of central cholinergic activity which is a key event in the biochemistry of AD.

The majority of the studies of accepted AD biomarkers to date have been carried out using samples of CSF obtained by lumbar puncture [10]. This is an invasive procedure that is particularly unpleasant for the subject and for which explicit consent is required. Recently, it has been shown that identification of blood biomarkers may allow the development of tests for AD [11,12]. In this study, we report that significant and reproducible levels of salivary Aβ_42 _can be detected in subjects and there is a specific correlation with development of AD pathology.

## Methods

### Subjects

The study included three groups: (1) 70 Alzheimer's disease (AD); (2) 56 elderly nondemented controls without neurological disease or cognitive impairment; and (3) 51 Parkinson's disease (PD) patients. All AD cases included in these series were diagnosed with dementia according to the Diagnostic and Statistical Manual of Mental Disorders (DSM)-IV criteria [13], and NINCDS-ADRDA criteria [14], and diagnosis required evidence of cognitive decline, (neuropsychological test battery, clinical mental examination) as well as evidence of impairment in social or occupational function. The mini-mental state examination (MMSE) was used to assess cognitive function [15]. The mean value of MMSE score for the AD patients was 17. All cases had an extensive biochemical measurement including levels of vitamin B_12 _and folate and thyroid hormones and neuroimaging techniques (brain MRI and/or CT scan). Classification of mild, moderate and severe degrees of AD was performed, and the diagnosis of vascular dementia was excluded in all cases, using DSM-III-R criteria. The control group was formed of family members or caregivers of the AD patients, who all had a clinical interview with a senior neurologist that showed a completely normal cognitive and functional level. No formal neuropsychological battery was performed in this group. PD group was formed of patients who had been diagnosed under the criteria of probable PD [16,17]. Demographic and health characteristics of the final sample (n = 136) are presented (table 1).

**Table 1:** Demographic and health characteristics of the final sample (n = 136)

	Mean age	Sex Men/Women	Mean MMSE (range)	Mean onset (range)
AD patients	77.20 (60-91)	21/49	17 (4-28)	2.56 (0-12)
PD patients	72.96 (60-93)	26/25	28 (22-30)	3.8 (1-5)
Control subjects	74.35 (64-85)	17/39	nd	

MMSE = mini-mental state examination; AD = Alzheimer's disease; PD = Parkinson's disease; nd = not determined.

### Saliva and blood collection

This study was approved by the Ethic Committee of Clinical Investigation of the Hospital '12 de Octubre'. Informed consent from all subjects was obtained prior to their participation Saliva samples were obtained from both healthy volunteers and patients with AD and PD and the study was carried out with full ethical permission. Saliva samples were collected in sterile plastic containers previously treated with 2% sodium azide solution, a concentration which has been shown to be sufficient to prevent microbial decomposition of saliva [18]. Participants were asked to wait at least 4 hours after eating or drinking (initiated at approximately the same time for each participant (13:00 hours), before providing saliva samples of approximately 1 ml into the containers, and these were centrifuged at 1500 rpm for 5 minutes to remove debris, in a similar manner to that previously described [19]. Then, the samples were immediately frozen at -80°C until used. Blood samples were obtained through antecubital vein puncture. Blood was centrifuged at 2500 rpm for 10 minutes, and plasma was collected, aliquoted and immediately frozen at -80°C. Cellular fraction was used for DNA extraction and genotyping assays.

### Apo E genotyping

Apo E genotypes of AD and control subjects were determined by established methods as described previously [20]. Genomic DNA was extracted from peripheral blood using Illustra™ blood genomicPrep Mini Spin Kit (GE Healthcare). Apo E genotyping (ε2/ε3/ε4 isoforms) was performed using FRET probes.

### Immunoassays

30 μl of saliva sample was mixed with an equal volume of 2× SDS sample buffer and denatured by heating at 95°C for 5 minutes. All samples were resolved by 10% SDS-polyacrylamide gel electrophoresis and transferred to nitrocellulose membranes (Bio-Rad) by electroblotting as previously described [21]. The following antibodies were used: mouse monoclonal anti-gelsolin (Sigma-Aldrich), rabbit polyclonal anti-TTR (Santa Cruz Biotechnology), goat anti-mouse HRP-conjugated (Bio-Rad), and goat anti-rabbit HRP-conjugated (Bio-Rad). Peroxidase-labeled lectin (Sigma) was used as gel loading control. Densitometric analysis was performed using ImageJ software (NIH Image).

Levels of human endogenous Aβ_40 _and Aβ_42 _in saliva and human plasma samples were determined with human specific enzyme-linked immunosorbent assay (ELISA) (Biosource International, Invitrogen), according to the manufacturer's instructions and as previously described [22]. 50 μl of saliva and plasma samples were added in duplicate to the microtiter wells. Detection limit of the assay was 6 pg/ml for Aβ_40 _and 1 pg/ml for Aβ_42_.

Previous to the immunoassays, protein concentration was assessed using a Spectrophotometer NanoDrop ND-1000, to normalize sample protein levels.

### Statistical analysis

Data were analysed with SPSS for Windows (version 15.0). To compare demographic, clinical, and saliva and plasma data between groups, we used ANOVA followed by a Tuckey-Kramer test, and Mann-Whitney *U*-test analysis when appropriate. The differences were considered to be significant at p < 0.05. The Spearman rank correlation was used for correlation analyses.

## Results

177 patients with AD, PD and aged controls were assessed, and Aβ_40 _and Aβ_42 _levels were measured in saliva from these patients using a sensitive and specific Aβ ELISA. It is particularly relevant that we were able to detect Aβ_40 _and Aβ_42 _in human saliva by a simple and reproducible method. The groups did not differ significantly by age or sex. Our findings show that saliva concentration of Aβ_42 _has a tendency to increase in AD patients compared with PD and control groups, but this effect was not statistically significant. Interestingly, when we analyzed the three categories of AD patients, we found that saliva Aβ_42 _levels were significantly increased in the first category of AD patients [in mild AD stage (p = 0.043, and table 2)], whereas saliva Aβ_42 _levels in moderate AD stage are also increased but with a high standard deviation (SD). Interestingly, the third category of AD, the severe AD stage, has similar than those observed in control group (table 2). Age range was similar between all AD stages and the control group. A 2 × 2 contingency table analysis, with a cut-off = 7.85 pg/ml, allowed the calculation of sensitivity and specificity (defined as the proportion of true positive and the portion of true negative that are correctly identified by the test, respectively). The results were 16% and 93%, respectively. In addition, we performed ROC curve analysis, with 0.547 (area under the curve) AUC (95% CI 0.4-0.68). On the other hand, Aβ_40 _was unchanged between AD patients and healthy subjects (table 2). We also analyzed the ratio between saliva Aβ_42 _and Aβ_40 _and we found that this ratio was higher, but not statistically significant (p = 0.2), in mild and moderate AD patients (0.35 and 0.54, respectively) in compared with control subject (0.13), whereas it was unchanged in severe AD patients (0.12).

**Table 2:** Saliva Aβ_42 _levels in patients with neurodegenerative diseases and control subjects

Group	Aβ_42 _(pg/ml)	Aβ_40 _(pg/ml)	No. of subjects
AD patients	**6.81 ± 20.04**	**22.3 ± 4.88**	70
Mild	7.67 ± 16.25*	21.87 ± 5.7	29
Moderate	11.70 ± 34.76	21.5 ± 4.17	24
Severe	3.03 ± 3.49	23.92 ± 4.55	17
PD patients	3.66 ± 4.21	26.41 ± 5.12	51
Control subjects	2.89 ± 4.96	20.82 ± 5.55	56

AD = Alzheimer's disease; PD = Parkinson's disease. Values are mean ± SD. *p < 0.05 versus control subjects

### Overall, saliva Aβ_42 _levels were not significantly higher with age

However, comparison of AD patients aged 60-65 with those aged 66-70 showed highly significant elevation in saliva Aβ_42 _levels (1.64 ± 0.44 pg/ml versus 6.46 ± 3.43 pg/ml, p = 0.016).

In addition, plasma levels of Aβ_40 _and Aβ_42 _did not differ significantly between patients with AD and control subjects (259 ± 91.9 pg/ml versus 225.1 ± 77.3 pg/ml, and 42.4 ± 92.7 pg/ml versus 52.4 ± 68.9 pg/ml, respectively), in accordance with recent studies [23]. Spearman rank analysis of plasma and saliva levels was not significant for either Aβ_40 _or Aβ_42 _levels.

To determine whether the elevated saliva Aβ_42 _was associated with the Apo E ε4 allele, all subjects were genotyped for ApoE and their Aβ_42 _levels were analyzed according to the ApoE genotypes. The Apo E ε4 allele frequencies were 45% (15 with Apo E ε3/4 genotype and 3 with Apo E ε4/4 genotype) in the AD group and 12% (3 with Apo E ε3/4 genotype and 1 with Apo E ε2/4 genotype) in the control group. Firstly, we found that ApoE genotype correlates with AD onset (Spearman rank correlation R = 0.428, p = 0.001). Levels of Aβ_42 _were higher, but not statistically significantly, in patients with AD and without the Apo E ε4 allele than in those with the allele (table 3). Levels of Aβ_42 _were similar in controls with and without Apo E ε4 allele (table 3).

**Table 3:** Saliva Aβ_42 _levels in patients with AD and control subjects

Group	No. Of subjects	Age	Aβ_42 _(pg/ml)
AD patients	38		
With Apo E ε4	18	75.18 ± 7.91	6.42 ± 15.48
Without Apo E ε4	20	75.05 ± 8.85	12.52 ± 33.58
Control subjects	34		
With Apo E ε4	4	68.5 ± 8.70	2.05 ± 1.48
Without Apo E ε4	30	69.10 ± 9.25	2.51 ± 2.61

Data are mean ± SD; AD = Alzheimer's disease.

**Figure 1:**
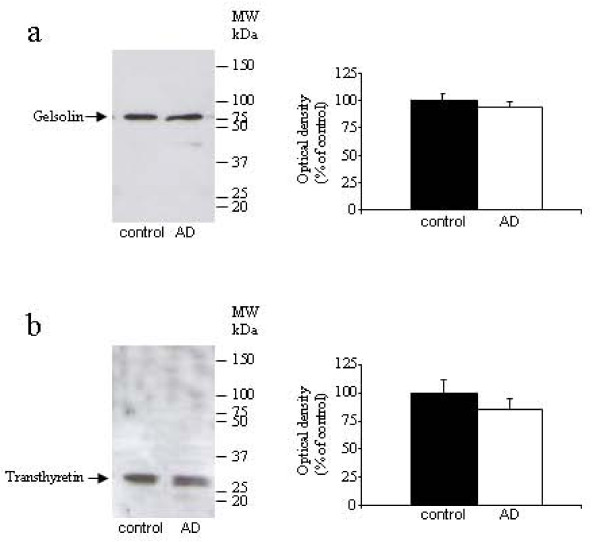
**Western-blot analysis of saliva levels of gelsolin (**a**) and transthyretin (**b**) in AD and control groups (n = 41-44 per group)**. Figures shows representative blots and quantitative from 4 independent measurements.

To test that protein changes are specific to Aβ_42 _levels, we measured protein concentration in saliva samples from all experimental groups. We did not find significant differences between groups (6.39 ± 3.68 ng/μl in aged subjects, 6.49 ± 4.79 ng/μl in AD patients, and 6.67 ± 5.71 ng/μl in PD patients, data are expressed as mean ± SEM). Additionally, we tested others protein concentrations in these saliva samples. We choose two proteins with Aβ-carrier function, gelsolin, and transthyretin. Using Western-blot assays, we did not observe an alteration in gelsolin (figure 1a) and transthyretin (figure 1b) expression in AD patients compared with control subjects. These results support our finding in human saliva that Aβ_42 _levels are closely associated with AD diagnosis.

Demographic risk factors could modify the association between pathological saliva Aβ_42 _and progression to AD. One of the studied variables was MMSE score. Mean MMSE score of AD patients was 17 (range 4-28) (table 1), and correlates with the three categories of AD (Spearman rank correlation R = -0.763, p = 0.001). The relationship between these variables was similar for males or females. Other studied risk factors in the AD group were: hypertension (with a prevalence of 47.1%), hypercholesterol (10%), arthritis (20%), depression (20%), diabetes (12.9%), and heart disease (15.7%). We analyzed whether there was an association between these risk factors and saliva Aβ_42 _levels. With Mann-Whitney *U*-test analysis, we found that the concentration of saliva Aβ_42 _correlates with sex (95% CI 0.71-2.29, p = 0.002) in the AD group.

## Discussion

Early diagnosis of AD represents a primary goal, and the role of biomarkers seems to be crucial in a routine clinical setting. The main findings of this study are that saliva Aβ_42 _levels are significantly elevated in early stage AD patients in comparison to control subjects, and we suggest that this effect is specific to AD and not to other neurodegenerative disorders, including PD. These data result in a slightly higher specificity, and suggest that measuring saliva Aβ_42 _may be used as biomarker to identify and confirm early AD diagnosis.

Biomarkers are required to improve the diagnostic sensitivity and specificity and to monitor the biological activity of AD in terms of the burden of neuronal involvement and the rate of disease progression. They must initially supplement our more traditional neuropsychological and imaging markers and may progress to provide useful tool to test the pharmacological action of anti-dementia compounds [24,25]. The currently best validated CSF biomarkers, pTau and Aβ_42_, with a reported sensitivity and specificity of around 90-95% for the diagnosis of AD, show generally a good correlation with cerebral tau and Aβ pathology [26-28]. As biomarkers in plasma several substances have been examined, but none of these markers had enough sensitivity or specificity to diagnose AD [24].

Saliva is frequently used to test for the levels of a number of hormones, and is considered as a non-invasive technique [29]. Therefore, the identification of robust and reproducible Aβ_42 _expression in saliva is of particular importance as it may serve as a potential indicator of AD neuropathology that can be measured with the minimum of stress for the subject. The mechanism by which Aβ_42 _accumulates in saliva is unclear. This localization could result from release of this peptide from salivary glands by APP processing as a consequence of secretase enzymes action in salivary epithelial cells [7]. Our data could reflect a similar situation to brain Aβ generation. The vast majority of studies performed so far have reported an increased accumulation of Aβ_42 _in cerebral parenchyma, mainly as senile plaques [30], whereas Aβ_42 _levels were reduced in CSF of patients with AD [31,32]. The significance of saliva Aβ levels in relation to Aβ accumulation in the brain is unknown; however, their concentrations are comparable with those observed from tissues other than brain, including lens [33]. Lower levels of CSF Aβ_42 _in the AD group may be explained due to loss of neurons that produce the APP, brain Aβ accumulation, and/or decreased Aβ clearance, as a consequence of impaired blood-brain barrier. As, the latter is not present in salivary glands, Aβ produced from APP glands may be delivered and accumulated in the saliva, mainly in mild AD patients, whereas, in severe AD stage, saliva Aβ_42 _levels return to control values. This pattern may reflect a parallelism with CSF Aβ_42 _levels. The association of different combinations of saliva Aβ levels with AD was independent of established risk factors such as age or Apo E genotype, major risk factors for sporadic AD. However, we found a significant correlation between sex and saliva Aβ_42 _levels in patients with AD. With several discrepancies, our data are consistent with previous studies using CSF and plasma samples [27,34]. Several studies had described no correlation between CSF Aβ levels and dementia severity [27,34-36]. However, others showed weak [37], or strong [38] correlation between these variables. In our study, we showed no significant relationship between saliva Aβ_42 _levels and MMSE scores, however when we analyzed the three categories of AD patients, accordingly with the functional capacity, we found a significant correlation. Thus, large and longitudinal studies with a greater number of samples will be necessary to determine conclusively whether there is a relationship between saliva Aβ_42 _levels and progression of AD.

Decreased saliva production is common amongst elderly people, and in patients with AD, salivary flow from the submandibular gland has been found to be significantly impaired [39]. However, the protein concentration of the saliva samples obtained from the elderly control subjects was similar to those of the subjects either with AD or PD. Additionally, we decided to investigate whether the concentration of other proteins may be changed in saliva samples from AD patients. Aβ forms complexes with protein carriers, including transthyretin or gelsolin, to prevent peptide polymerization and aggregation [40-42]. Since these proteins are present in body fluids [32,43], and are significantly reduced in CSF from AD patients [32,44,45], we chose them to test our hypothesis. Since total protein concentration was similar in AD patients and healthy subjects, and these secreted proteins was unchanged in both groups, we may suggest that elevated Aβ_42 _levels represent a specific change for AD, and can not be attributed to a general increase in salivary protein concentration.

As usual, there were various difficulties in the measurement of Aβ levels in body fluids. Additionally, Aβ values decreased over time, even if the samples were frozen [46]. Although range of saliva Aβ_42 _levels was high, we consider that is not in discrepancy with those reported in plasma or CSF samples from AD patients, with range seen from 100 pg/ml to 770 pg/ml, and 25 pg/ml to 325 pg/ml, respectively [34]. In this study, we demonstrate the remarkable reproducibility of the saliva Aβ ELISA in different series of repetitive measurements. It is particularly significant that saliva analyses of Aβ_42 _are increased in mild AD patients, whereas in the severe stage, associated with a greater neurodegeneration, the levels are unchanged. Our data was supported by the ratio Aβ_42 _/Aβ_40 _in saliva that was higher in mild and moderate AD patients in compared with control subject, whereas it was unchanged in severe AD patients. This fact is consistent with the main objective of AD biomarkers: the early diagnosis. Indeed, further studies will be performed to determine saliva Aβ_42 _levels in mild cognitive impairment (MCI), an intermediate stage to dementia, how these levels change across the transition from normal to MCI, and the interactions with age and Apo E genotype. If validated in other consecutive studies with long follow-up and large number of patients, these results may have an effect on diagnosis and on the design of clinical trials of patients with mild AD.

## Conclusions

There were no significant differences in Aβ_42 _levels between AD, PD and controls subjects. However, our findings show that saliva concentration of Aβ_42 _differs between mild AD and non-demented control subjects, and that this is a specific characteristic of AD, being absent in PD. To our knowledge, no study to date has investigated the possibility of overlapping AD-associated Aβ levels in saliva and brain of subjects with this disorder. Our results show that saliva analyses of Aβ_42 _are powerful risk markers for development of clinical AD in patients.

## Authors' contributions

FB-P: conceived of the study, provide clinical samples and diagnostic data, and participated in its design and coordination.

DA: carried out the immunoassays and performed the statistical analysis.

TV: carried out the molecular genetic studies.

JAM: provide clinical samples and diagnostic data.

EC: conceived of the study, participated in its design and coordination, and wrote the manuscript.

All authors read and approved the final manuscript.

## Pre-publication history

The pre-publication history for this paper can be accessed here:

http://www.biomedcentral.com/1471-2377/10/108/prepub
